# Shared decision-making in pediatric physical therapy: A qualitative study among adolescents, parents, and physical therapists

**DOI:** 10.1371/journal.pone.0352677

**Published:** 2026-06-30

**Authors:** Selina Limmen, Dorinde L. Korteling, Manon A. T. Bloemen, Michiel A. J. Luijten, Marjolijn Ketelaar, Raoul H. H. Engelbert, Dirk T. Ubbink, Marij A. Hillen, Eugene A. A. Rameckers, Hedy A. van Oers, Lotte Haverman

**Affiliations:** 1 Department of Child and Adolescent Psychiatry & Psychosocial Care, Amsterdam UMC, location University of Amsterdam, Emma Children’s Hospital, Amsterdam, the Netherlands; 2 Child development, Amsterdam Reproduction and Development, Amsterdam, the Netherlands; 3 Mental health, Amsterdam Public Health, Amsterdam, the Netherlands; 4 Personalized Medicine, Amsterdam Public Health, Amsterdam, the Netherlands; 5 Methodology, Amsterdam Public Health, Amsterdam, the Netherlands; 6 Research Group Moving, Growing and Thriving Together, HU University of Applied Sciences Utrecht, Utrecht, the Netherlands; 7 Department of Epidemiology and Data Science, Amsterdam UMC, Vrije Universiteit, Amsterdam, the Netherlands; 8 UMC Utrecht Brain Center, University Medical Center Utrecht, Utrecht, the Netherlands; 9 Center of Excellence for Rehabilitation Medicine Utrecht, De Hoogstraat Rehabilitation, Utrecht, The Netherlands; 10 Department of Rehabilitation Medicine, Amsterdam Movement Sciences, Amsterdam UMC, location University of Amsterdam, Amsterdam, the Netherlands; 11 Centre of Expertise Urban Vitality, Faculty of Health, Amsterdam University of Applied Sciences, Amsterdam, the Netherlands; 12 Department of Surgery, Amsterdam UMC, location University of Amsterdam, Amsterdam, The Netherlands; 13 Quality of Care, Amsterdam Public Health, Amsterdam, the Netherlands; 14 Department of Medical Psychology, Amsterdam UMC, location University of Amsterdam, Amsterdam, the Netherlands; 15 Department of rehabilitation, CAPHRI, Maastricht University, Maastricht, the Netherlands; 16 Centre of Expertise, Adelante Rehabilitation Centre, Valkenburg, the Netherlands; 17 Rehabilitation Science and Physiotherapy, REVAL, Hasselt University, Hasselt, Belgium; 18 Amsterdam Public Health, Digital health, Amsterdam, the Netherlands; Cairo University, EGYPT

## Abstract

**Objective:**

Shared Decision Making (SDM) is a collaborative process between patients and clinicians. A structured approach for SDM in pediatric physical therapy is lacking. This two-phase study aimed to 1) explore how and when to apply SDM in pediatric physical therapy in primary healthcare, and to identify barriers and facilitators influencing its use, and 2) adapt an SDM model for use in pediatric physical therapy and propose strategies for implementation.

**Methods:**

The study consisted of two phases. In Phase 1, six focus groups were conducted, two per participant group: adolescents (12-18y, n = 11), parents of children (4-18y, n = 9), and pediatric physical therapists (n = 6). A qualitative survey among 46 pediatric physical therapists validated focus group results. An inductive analysis explored how and when SDM should be applied, and a deductive analysis identified barriers and facilitators by linking codes to Consolidated Framework for Implementation Research (CFIR) domains. In Phase 2, the research team integrated results into an existing goal-based SDM-model, and implementation strategies were selected using the CFIR-Expert Recommendations for Implementing Change tool.

**Results:**

SDM can begin at intake and goal setting, with ongoing, individualized involvement of children and parents throughout therapy. When comparing therapy options, treatment frequency, duration, homework, expectations, and possibilities at home can be discussed. Barriers included time constraints and the challenge of balancing multiple perspectives, while facilitators were the possibility to adapt SDM conversations per family and a supportive practice culture. A goal-based SDM-model was adapted for pediatric physical therapy. Implementation strategies identified were professional training, use of SDM tools, sufficient contact with parents, time to learn SDM, a supportive team culture, and empowering parents and children.

**Discussion:**

This study provides guidelines for implementing SDM in pediatric physical therapy in primary care. A multifaceted implementation approach, guided by this study’s implementation strategies, may enhance SDM integration into clinical practice.

## Introduction

Pediatric physical therapy addresses movement-related challenges in children with a wide range of conditions, including developmental delays, neurological disorders, musculoskeletal issues, and congenital abnormalities [1,2]. Therapy is tailored to goals formulated by children and parents and often includes play-based activities to promote independent functioning and participation in daily life [2,3]. In the Netherlands, primary care physical therapy refers to direct-access outpatient services that act as the first point of contact in the healthcare system; children may be referred or seen without referral. Services are mostly delivered in private practices but may also occur at home or school. A typical pediatric physical therapy trajectory includes an intake with interview, examination, goal setting and therapy planning, followed by weekly individual in-clinic sessions complemented by a home exercise program and periodic evaluations. In the Netherlands, 18 sessions are covered under basic health insurance, while children with chronic conditions have access to unlimited sessions.

Shared decision-making (SDM) was recently incorporated into the Dutch competency profile of the pediatric physical therapist (PPT) [3]. SDM is a collaborative process in which healthcare professionals and patients jointly make decisions about care [4,5]. It is recognized in healthcare as a generic approach to improve quality of care, and is widely recommended by professionals and policy makers [6,7]. Within physical therapy, SDM enables tailored care, accurate treatment expectations, increased treatment satisfaction, therapy adherence, and overall well-being in patients [8–10]. Most adult patients undergoing physical therapy desire active involvement in decision-making [9]. However, information on SDM in pediatric physical therapy is limited. Globally recognized SDM models include informing patients of joint decision-making, discussing options and preferences, and reaching agreement [4,5]. These models, however, were largely designed for single conditions with limited preference-sensitive options [11].

Research on SDM in physical therapy has mainly focused on adults with single musculoskeletal conditions [8,9]. Knowledge of SDM in pediatric physical therapy is limited. Pediatric physical therapy often involves children with multiple movement-related challenges, resulting in several therapy goals. A goal-based SDM approach, centering on the patient’s most pressing health issues [11,12], seems most suitable. Van der Pol et al. developed such a model for older patients with multifactorial issues, distinguishing six phases: 1) ‘Preparation’, history/ problem analysis, 2) ‘Goal talk’, identify goals and discussion partner (patient/ caregiver), 3) ‘Choice talk’, offer choice, 4) ‘Option talk’, list treatment options, 5) ‘Decision talk’, discuss preferences and decide, and 6) ‘Evaluation’, evaluate and prepare treatment plan.^8^ Besides its focus on personal goals, another advantage of using the model of van der Pol et al.^11^ in pediatric physical therapy is the inclusion of caregivers. In the Netherlands, caregiver consent is mandatory under age 16 [13]. Caregivers may have different values or preferences than their child that need to be considered.

The model of van der Pol et al., although valuable, was originally developed for older patients and does not account for child involvement. Child involvement in SDM exists along a developmental spectrum, with their capacity and autonomy increasing over time. Correspondingly, the role of parents gradually diminishes as the child matures and becomes more capable of independent judgment [14,15]. The limited literature on pediatric SDM primarily concerns hospital settings [14,16], involving high-stake decisions in complex or life-threatening conditions [17]. These studies mainly focus on the inclusion of parents in SDM [18]. In contrast, pediatric physical therapy in a primary care generally involves low-risk, adaptable therapy focused on functional improvement and participation rather than life-threatening outcomes. A study in England, involving children with cerebral palsy, explored experiences of parents, children, and PPTs regarding SDM in community-based pediatric physical therapy. Involvement of different parties varied depending on the type of complaint, but child involvement was consistently limited [19]. Authors recommended greater clarity about roles of involved parties and types of decisions suitable for SDM [19].

Despite growing endorsement of SDM, practical, context-specific guidance and evidence-informed implementation strategies for pediatric physical therapy remain scarce. Therefore, this study aims to 1) explore how and when SDM should be applied in pediatric physical therapy, and identify barriers and facilitators influencing its use, and 2) develop a context-specific SDM model for pediatric physical therapy and propose strategies for its implementation.

## Methods

**Fig 1:**
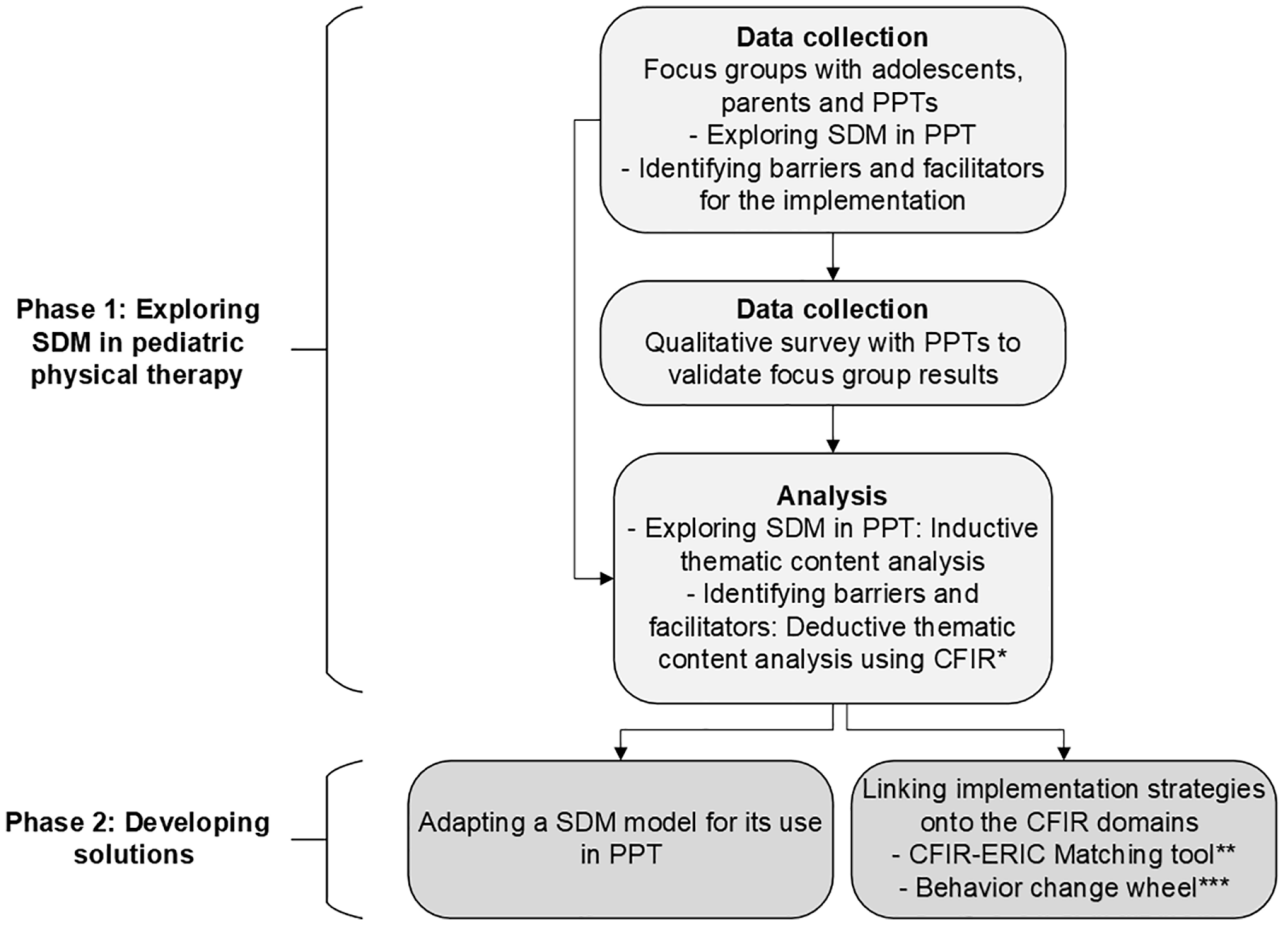
Visual presentation of the methods. SDM = Shared decision-making; PPT = Pediatric physical therapy; *Damschroder et al. 2022 [21]; **CFIR Research Team-Center for Clinical Management Research. 2025 [22]; ***Michie et al. 2011 [23].

We conducted a multimethod qualitative study and reported it according to the Standards for Reporting Qualitative Research (SRQR), provided in Supplementary file 1 [20]. The study was designed in two consecutive phases. Phase 1 focused on exploring how and when SDM should be applied in pediatric physical therapy and identifying barriers and facilitators to its implementation through focus groups and a qualitative survey. Phase 2 focused on developing solutions by integrating phase 1 findings into the goal-based SDM model of van der Pol [11] and identifying strategies for implementation. An overview of the study design is presented in Fig 1. The Medical Ethics Review Board of the Amsterdam UMC, location AMC waived the requirement for approval under the Dutch Law on research with humans (W23_005#23.026).

### Phase 1: Exploring SDM in pediatric physical therapy

#### Focus groups.

**Participants:** To explore the perspectives of the involved parties in SDM, six focus groups were conducted between June 30, 2023, and March 5, 2024, with two focus groups per participant category: 1) parents of children aged 4–18 years receiving pediatric physical therapy, 2) adolescents aged 12–18 years receiving pediatric physical therapy, and 3) registered PPTs working in primary healthcare. Participant groups were separated to facilitate a safe environment for open expression. Children 12 years or older were included as adolescents from than age are generally capable of abstract reasoning, hypothetical thinking, and articulating perspectives on complex concepts such as SDM [24]. We utilized purposive sampling including a variety of ages, conditions, and level of physical functioning of children in the parents and adolescents group and work experience in PPTs. This was supplemented with convenience sampling [25]. Parents, adolescents, and PPTs were recruited through pediatric physical therapy practices in the Netherlands. Additionally, parents and adolescents were recruited via social media networks of patient organizations, and PPTs through personal networks and a call on the platform of the Dutch association for pediatric physical therapy (NVFK). All participants provided written informed consent. For adolescents <16 years, written assent was obtained alongside written consent from their caregiver. Each participant received a €15 gift card.

**Data collection:** The focus groups each included three to eight participants and lasted 120 minutes. A moderator (SL) experienced in qualitative research and pediatric physical therapy and co-moderator (DK, ML or HO) facilitated the sessions. Focus groups were conducted face-to-face at physical therapy practices located throughout the Netherlands. One focus group was held online for parents who were not able to visit a face-to-face session, and one for PPTs due to the geographically dispersed nature of participants across the Netherlands. One week prior to the focus groups, participants completed a sensitizing assignment at home, encouraging them to reflect on decision-making via a timeline related to pediatric physical therapy on which participants could write, draw, or select emoticons. Sensitization helps participants reflect on relevant past experiences to share during the focus groups, for example by using a patient journey [26,27]. The sensitizer and focus group topic list were developed by SL and HO in close collaboration with SDM experts (DU, MH) and subsequently reviewed with the full author team, including experts in pediatric physical therapy and psychology. The pilot-tested topic list included a discussion of the sensitizer and participants’ perceptions of SDM, including their views on the provided definition [4,5], with the aim of establishing a common understanding of SDM. Participants then shared their experiences with SDM, followed by a discussion on how and when SDM should be applied in pediatric physical therapy. Finally, barriers and facilitators to SDM implementation were explored. All focus groups were audio-recorded. Sociodemographic information of all participants was collected with a questionnaire directly after the focus groups. To get an indication on the varying levels of functional mobility of our participants, we used Functional Mobility Scale [28]. This 6-level scale classifies children’s functional mobility based on mobility aid use and is validated for children with cerebral palsy [29]. The FMS was completed by adolescents and parents focusing on the child receiving PPT.

**Analysis:** All documents were pseudonymized, with participant information secured in a coded identification log. Focus group results were analyzed with thematic content analysis using MaxQDA [30]. Recordings were transcribed verbatim using ‘WordOnline’ (live sessions), and ‘Microsoft Teams’ (online sessions). Transcripts were checked by a master’s degree pediatric physical therapy student (WD or FH). Analyses were conducted by a researcher (SL) and a student (WD) [31]. Exploring how and when SDM should be applied was analyzed with an inductive approach. First, text fragments were open-coded into concepts. Half of the transcripts were open-coded by two researchers independently (SL & WD) and compared until consensus was reached. The remaining transcripts were open-coded by WD, and checked randomly by SL. SL and WD jointly categorized open codes into initial themes, which were then reviewed and refined into final themes, and placed in order of where they naturally occur in daily practice. Final themes were discussed in depth with SL and HO until consensus was reached.

Identification of barriers and facilitators was analyzed with a deductive approach. SL linked text fragments to preexisting constructs within the domains of the updated version of the Consolidated Framework for Implementation Research (CFIR) [21]. CFIR provides a pragmatic structure for identifying potential influences on implementation [21]. Codes were discussed in depth with HO until consensus was reached. CFIR domains included in this study were ‘innovation’ (SDM), ‘outer setting’ (external context outside physical therapy practices), ‘inner setting’ (pediatric physical therapy practices), and ‘individuals’ (individuals involved in the implementation).

#### Survey.

**Participants:** A qualitative survey was conducted to validate the focus group results. Survey participants included registered PPTs working in primary care throughout the Netherlands. The qualitative survey was distributed between March 22 and July 3, 2024, to 51 PPTs before they participated in a training on SDM and the use of Patient-Reported Outcome Measures (PROMs) in pediatric physical therapy. Participants came from 12 pediatric physical therapy practices who voluntarily registered for this training, which was organized and provided by the project group. Informed consent was obtained.

**Data collection:** Focus group results were summarized into a PDF-file and videoclip, and presented to the PPTs via email prior to the training. Subsequently, three open questions were asked through a survey sent by email: 1) ‘*What does SDM in pediatric physical therapy mean to you?’,* 2) *‘Does your vision align with the provided information on SDM in pediatric physical therapy? If not, please explain’* and 3) *‘Can you identify with the provided barriers and facilitators? Do you have any additions?’*

**Analyses:** Survey results were coded according to the same principles used for the focus groups. Open codes were placed in the existing code-trees by SL, checked by HO, and discussed in depth by SL and HO together until consensus was reached. If open codes could not be placed into the existing code threes, a new theme was created.

### Phase 2: Developing solutions

#### Adapting an SDM model for its use in pediatric physical therapy.

Phase 1 results were integrated into the existing goal-based SDM model developed by van der Pol et al. [11], which was chosen because it is organized around jointly formulated goals of patients and caregivers and acknowledges multiple discussion partners, while remaining conceptually consistent with widely used SDM models, such as those proposed by Elwyn [4] and Stiggelbout [5]. Themes from the focus groups were linked to the stages of the model by SL, based on where they naturally occurred in practice. For example, discussions about mutual expectations, and who initiated therapy were mapped onto the *Preparation* phase. Afterwards, SL discussed this in depth with HO and LH. The results were Subsequently presented to the research team for discussion, and once consensus was reached, additional comments were incorporated into the model. The research team consisted of researchers experienced in PPT (n = 4), SDM (n = 2), psychology (n = 3), neuroscience (n = 1), and rehabilitation and patient involvement (n = 1).

#### Matching implementation strategies.

As an SDM model alone does not address all barriers to implementing SDM in PPT, we also identified multifaceted implementation strategies. The updated CFIR-ERIC (Expert Recommendations for Implementing Change) Strategy Matching File was used to match implementation strategies with the identified barriers and facilitators [22]. The CFIR-ERIC tool, developed by implementation researchers and clinicians, provides implementation strategies to reduce specific barriers based on the CFIR framework [32,33]. However, the CFIR-ERIC tool does not address specific individual-level components such as capability or motivation. In the updated CFIR framework, domains from the Capability, Motivation, Opportunity – Behavior (COM-B) model are integrated into the domain ‘individuals*’*. To be able to find strategies for barriers and facilitators within the domain ‘individuals*’* of CFIR, we supplemented the CFIR-ERIC strategies with those from the behavior change wheel, a framework providing strategies based on the COM-B[23]. Strategies from both frameworks were tailored to the context of pediatric physical therapy by SL and HO and discussed with LH until consensus was reached.

## Results

### Phase 1: Exploring SDM in pediatric physical therapy

Eleven adolescents, nine parents and six PPTs participated in the focus groups. Their characteristics are shown in Table 1. In addition, 46 PPTs completed the qualitative survey (response rate = 90%), of whom 71% fully agreed with the focus group findings and 29% provided additional perspectives or critical remarks.

**Table 1:** Sociodemographic information of focus group participants.

Sociodemographic information	Adolescents (n = 11)								Parents (n = 9)								PPTs (n = 6)
Age in years																	
Mean [SD]	12.9 [2.8]								45.4 (8.9)								45.3 (15.6)
Range	12-18								33-64								30-66
Gender																	
Male	3 (27.3%)								0								2 (33.3%)
Female	8 (72.7%)								9 (100%)								4 (66.7%)
Highest educational level*																	
Lower secondary	4 (36.4%)								0								0
Upper secondary	4 (36.4%)								1 (11.1%)								0
Post secondary non-tertiary	1 (9.1%)								3 (33.3%)								0
Bachelor	2 (18.2%)								3 (33.3%)								0
Master or doctoral	0								2 (22.2%)								6 (100%)
Duration of therapy in months																	
Median (IQR)	3 (1-12)								66 (15-72)								N/A
Range	0.5-108								4-96								N/A
Functional mobility scale of child treated by PPT**																	
	N	C	1	2	3	4	5	6	N	C	1	2	3	4	5	6	
Range 5 meters (n)								11		1	1	1				6	N/A
Range 50 meters (n)							1	10	1		1	1			2	4	N/A
Range 500 meters (n)							2	9	1		1	1			2	4	N/A
Work experience as a PPT in years																	
Median (IQR)	N/A								N/A								8.5 (3.8-22)
Range	N/A								N/A								3-43

PPT = Pediatric physical therapist; IQR = Inter-Quartile Range.

*Parents and PPTs highest and completed level of education. Adolescents’ current education (categorized according to International Standard Classification of Education (ISCED levels)).

** Functional mobility scale scores: “C” = crawling (for mobility at home), “N” = child does not complete the distance, 1 = uses wheelchair, 2 = uses a walker or frame, 3 = uses crutches, 4 = uses sticks (one or two), 5 = independent on LEVEL surfaces, 6 = independent on ALL surfaces.

#### How and when SDM can be applied.

Four themes were generated by the research team based on the focus groups and survey. Quotes are presented in Table 2.

**Table 2:** Quotes regarding aim one.

Quote	Quote
A	‘My goal was to be able to wear heels to prom in high school. I did not care about anything else. So, we worked hard towards that. That really motivated me.’ – Adolescent
B	‘The initial focus of the PPT should be to listen to my son and what he wants. My role as a parent is to add a bit more perspective. But it is really a team effort between the three of us.’ – Parent
C	‘It is very important that the PPT knows our home situation. For us it can be very complex, we have other children and a lot of appointments with other healthcare professionals. We do not always have time to do exercises at home.’ – Parent
D	‘Sometimes, I feel like a terrible mother when the PPT tells me what I need to do at home. I think, you have all the time, peace, and space here, but at home we have more children and I do not have your equipment.’ – Parent
E	‘As a PPT you provide a couple of games or exercises and the child gets to choose which particular game they want to play today.’ – PPT focus group
F	‘I had to go to physio twice a week, including Fridays, which totally messed up my hair-washing schedule for going out. So, I ended up doing nothing because I did not want to sweat. Eventually, we decided during an evaluation that I would go to the gym once a week instead, in addition to once a week pediatric physical therapy. That is going really well.’ – Adolescent

SDM = shared decision-making; PPT = pediatric physical therapist.

**Intake and goal setting:** PPTs and parents suggested that preparation for SDM in PPT starts at the intake where mutual expectations, perspectives, and the reason for therapy (including who initiated this) can be discussed. All subgroups emphasized the importance of including goal setting in SDM. PPTs and adolescents indicated that a clearly defined, jointly established goal can improve motivation for therapy (quote A), therapy adherence, and a benchmark for continuing or ending therapy. Some survey participants expressed their wish for practical tools guiding goal setting.

**Involvement of parents and children:** All subgroups suggested that children’s involvement should be as much as possible (quote B) and evaluated and discussed on an individual basis, based on characteristics such as age and developmental stage. Adolescents and parents preferred being asked if and how they wish to participate in decisions. Survey participants mentioned that during SDM, the focus is often too much on parents instead of children.

**Choosing a therapy plan:** When discussing therapy options, participants suggested to consider expected therapy period, frequency of meetings, home program, possible changes in behavior, and what can be expected from family and PPT. Parents and PPTs emphasized to address possibilities at home (e.g., resources, time, space) as a home program is often part of pediatric physical therapy (quote C and D).

**Occurrence of SDM throughout therapy:** Participants noted that SDM can occur at multiple stages during pediatric physical therapy. PPTs and adolescents suggested involving children in customizing exercises through SDM (quote E). All participant groups mentioned applying SDM during evaluations. They spoke of two evaluation types: 1) short, informal throughout every session, and 2) predetermined periodic moments. Examples of SDM topics in evaluations were setting new sub-goals and discussing feasibility of therapy (quote F). Last, parents and PPTs said SDM could help decide if a child needs referral to other (healthcare) professionals.

#### Barriers and facilitators towards the implementation of SDM in pediatric physical therapy.

Focus groups and survey results were placed within CFIR domains. Quotes are presented in Table 3.

**Table 3:** CFIR Domains and indicating barrier or facilitator and quotes.

CFIR domains	Quote
I. INNOVATION DOMAIN	
Adaptability of SDM (F)	A) ‘Even with the same condition, there are so many different factors involved in each case. Therefore, every SDM conversation is unique.’ – PPT survey B) ‘Imagine you have a child with an ankle sprain who recovers relatively quickly after a few consultations. Of course, you would apply SDM, but I believe SDM is more comprehensive for children with a chronic condition.’ – PPT focus group
Complexity of SDM (B)	C) ‘Divorced parents can be a big problem for optimal SDM if opinions do not align. It can be really challenging when you have to communicate with both. It takes a lot of time.’ – PPT focus group D) ‘I find it challenging when a patient does not want an evidence-based treatment, like massage. How do you handle that?’ – PPT survey
II. OUTER SETTING DOMAIN	
Policies and laws/finance (B)	E) ‘The role of the PPT is changing. Health insurances need to ensure that these services are covered financially, right? However, they are still lagging behind on this. So, for now, it remains a gray area.’ – PPT focus group
Partnership and connections (B/F)	F) ‘You should include the whole network around the child. As a parent, you do not necessarily have to coordinate with everyone. The PPT can act as the link between them.’ – Parent G) ‘In a rehabilitation center, all care is in one place. In primary care it is often spread out. You are not always aware of what other healthcare professionals are doing. While collaboration is possible, it can be challenging to establish due to factors like time and accessibility.’ – PPT survey
III. INNER SETTING DOMAIN	
Culture (B/F)	H) ‘I believe a key facilitator is when colleagues and employers consistently encourage one another to adopt SDM through ongoing conversations.’ – PPT survey
Physical infrastructure (B/F)	I) ‘My child used to receive physical therapy at a distant school. I often did not know what was happening during sessions, which I found disappointing. The therapist did not avoid contact, but since I was not there… Sometimes she forgot to update me or I forgot to ask, and weeks would pass. Now that therapy is at the practice, I am much more involved.’ – Parent
Work infrastructure (B/F)	J) ‘This practice exclusively employs revenue-based PPTs. We are not paid for the time we spend providing explanations or for administration. We feel like we have less time to implement new things due to financial pressure.’ – PPT survey K) ‘In our practice, the first appointment lasts one hour instead of half an hour. This generally provides enough time to conduct a thorough assessment, and apply SDM.’ – PPT survey
IV. INDIVIDUALS DOMAIN	
Innovation deliverers (PPTs) – Capability and motivation (B/F)	L) ‘They said, ‘He has DCD. You need to work with goals.’ I thought, ‘Okay, but where do we go from here?’ I had no information, so I had no idea how to decide on a therapy plan. I looked up what DCD meant, and I was quite shocked. I felt a bit alone with it all.’ – Parent M) ‘My therapist initially presented a treatment plan that I felt was too frequent and would prevent me from spending time with my friends. After discussing my concerns with my mom, she spoke to the therapist, and only then did we decided together on an alternative treatment plan.’ – Adolescent N) ‘Sometimes the input from the patient and the parent becomes too dominant in the process, and, at least for me, it feels like we are no longer seen as the specialist at some point.’ – PPT survey O) ‘SDM is a vision we are striving for, but in practice, we are still far from achieving it.’ – PPT survey
Innovation recipients (parents and children) – Capability and motivation (B/F)	P) ‘In the beginning, it is reassuring when the therapist takes the lead. Before we had a diagnosis, everything felt chaotic and we did not know where to start or what to expect. At that stage, it is nice to have an expert to guide you and help you focus, leading you step by step through the necessary choices.’ – Parent Q) ‘With cultural differences and low health literacy, I notice that parents often hand over the therapy entirely, and the child tends to see you as a more authoritative figure. In children with a Dutch cultural background and parents with higher health literacy, I find that parents are more likely to engage in SDM.’ – PPT focus group

F = facilitator; B = barrier; SDM = shared decision-making; PPT = pediatric physical therapist.

**Innovation (SDM):** The adaptability of SDM per family, based on for example number of therapy goals and complexity of the home environment, was seen as a facilitator by PPTs and adolescents (quote A & B). Nevertheless, PPTs found SDM challenging when interests of the therapist, child, and parents diverged, such as with divorced parents, cultural differences, or requests for non-evidence-based treatment (Quotes C–D). This was perceived as a barrier to its implementation due to uncertainty about how to appropriately manage these situations. In contrast to the focus groups, some PPTs mentioned in the survey that different opinions were not a barrier but a reason to apply SDM. Finally, the recurring presence of SDM throughout therapy was perceived as challenging by PPTs, as PPTs often forget to schedule pre-determined evaluation moments where SDM could be applied.

**Outer setting (external context):** Time constraints were repeatedly mentioned by PPTs as a barrier for implementing SDM and was said to be part of a broader problem within pediatric physical therapy. PPTs mentioned that multiple parts of the profession are changing, such as working in the environment of children and increasing administrative tasks (quote E). As a result, they felt constrained in their ability to adopt new approaches such as SDM. Parents saw collaboration between PPTs and other professionals as a facilitator for SDM, leading to well-coordinated care plans (quote F). PPTs acknowledged the value of such collaboration but cited limited time and awareness of other professionals’ availability and area of expertise as barriers (quote G). Some PPTs highlighted that involving other professionals could complicate SDM as opinions do not always align.

**Inner setting (physical therapy practices):** A practice culture encouraging open discussion and reflection on SDM in patient cases among colleagues was identified by PPTs as an important facilitator (quote H). PPTs and parents indicated that working in settings where parents are not regularly present, such as distant schools, hindered parental involvement in SDM (quote I). Home therapy was perceived as a facilitator for SDM by parents and PPTs, as it allows PPTs to gain better understanding of the family’s available resources and contextual factors, thereby enabling a more tailored care plan. PPTs mentioned lack of time as a barrier, as some PPTs work on a revenue basis (paid per patient), while others receive a fixed amount per month. Revenue-based models often put pressure on PPTs to consult with more patients daily, creating a rushed atmosphere (quote J). Furthermore, some practices provide paid administration time for PPTs where other practices do not (quote K). PPTs noted needing time to become familiar with new approaches, such as SDM, to effectively implement them.

**Individuals: Innovation deliverers (PPTs):** Variability in PPTs capabilities to communicate and involve families was frequently mentioned by all participant groups. Parents and adolescents reported that the PPT’s ability to effectively inform parents and children regarding the condition and the therapy options and tone of the therapist strongly influenced their sense of partnership (quote L). They mentioned that pictures, drawings, or videos, could support verbal information. Adolescents and parents noted that not all PPTs apply SDM, citing examples in which they were not actively involved in decisions (quote M). While most PPTs expressed high motivation to use SDM, some worried that parents’ preferences might at times overshadow professional expertise (quote N). Most PPTs mentioned that SDM comes naturally to them while some survey participants noted that the nature of their profession is more focused on ‘doing’ rather than ‘talking,’ putting SDM somewhat outside their comfort zone. Some PPTs mentioned that SDM is a new way of thinking and although their motivation is high, it will take time for them to adjust (quote O).

**Individuals: Innovation recipients (parents and children):** According to PPTs, characteristics such as low health literacy or intellectual disability could form a barrier for the capability of parents and children to participate in SDM. Some parents and adolescents found the start of therapy unfamiliar, which led them to place the responsibility for decision making on the PPT (quote P). After families became more familiar with therapy, they expressed a desire for greater involvement. With this, they emphasized the importance of regularly evaluating child and parent involvement in decision-making. Furthermore, PPTs mentioned that cultural aspects and beliefs of children and parents influence their motivation to be involved in SDM (quote Q). Last, PPTs mentioned that parents and children sometimes encounter incorrect information, for example on social media, making them less receptive to the PPT’s expertise during SDM. Survey participants suggested to give the child enough time and space to make a decision to enhance capability of the child to participate in decisions.

### Phase 2: Developing solutions

#### Adapted SDM model for its use in PPT.

**Fig 2:**
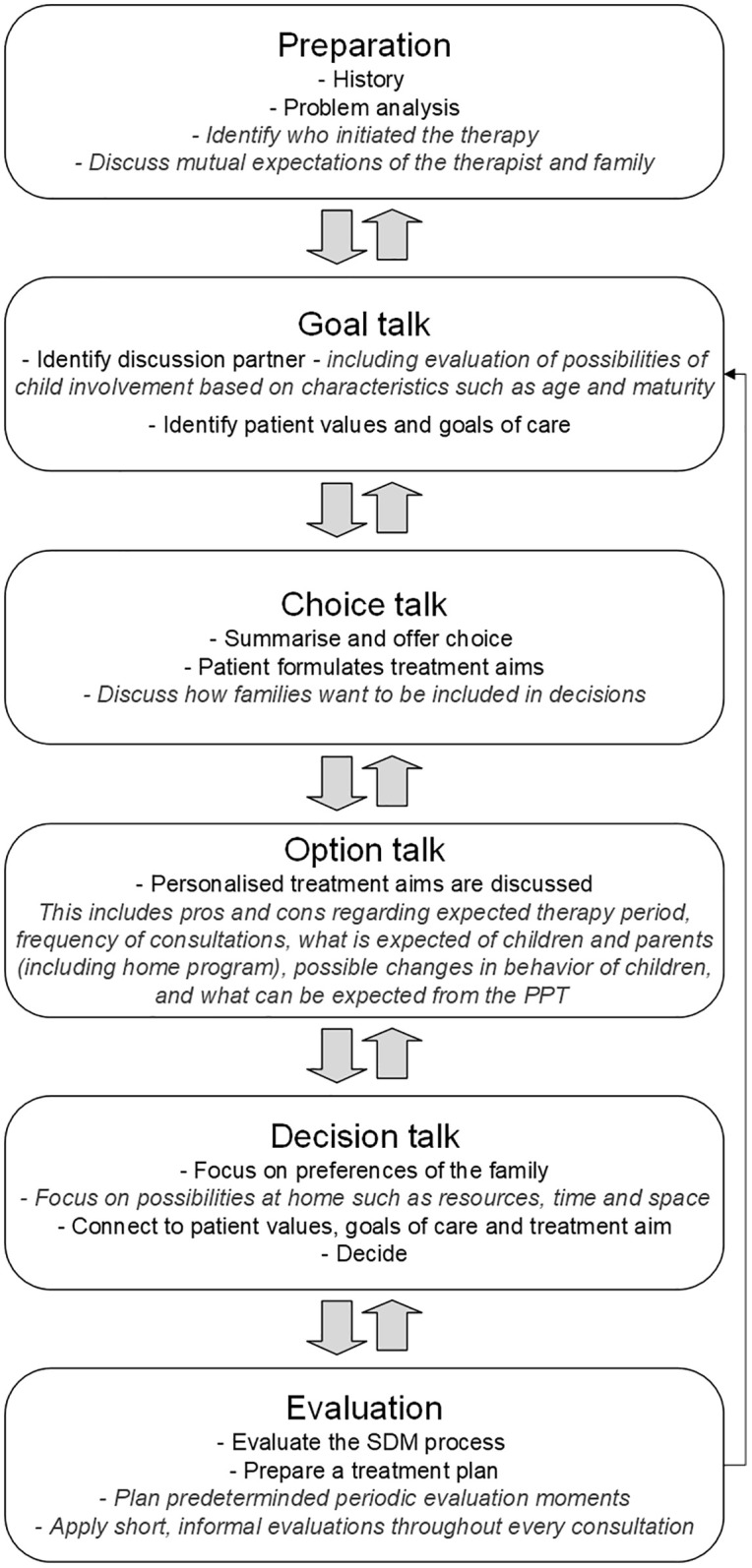
Additions to the goal-based shared decision-making model of van der Pol et al. [11] for pediatric physical therapy. *Additions specific for PPT in gray and Italic.* SDM = shared decision-making; PPT = pediatric physical therapist.

Qualitative study results were integrated into the goal-based SDM model of van der Pol et al. [11], resulting in an adapted SDM model for PPT (Fig 2). The model can be used across different therapy locations, including private practices, home-based therapy, and school locations. The model includes the child, parent and PPT. It focuses on decisions regarding the therapy plan and can be applied both at the start of therapy and during evaluation moments. Although our qualitative study indicated that SDM may also inform the selection of individual exercises, the model does not focus on this, as such shared decisions are made more rapidly and do not require all model-steps. While collaboration with other professionals was identified as a facilitator for aligning care plans, this was not incorporated into the model because not all children receive multidisciplinary care. As the qualitative study indicated the importance of giving children adequate time and space to form decisions, the model does not need to be completed within a single consultation; the pacing of SDM conversations can be adapted to family preferences. When necessary, such as in families with lower health literacy, the therapist may take a more guiding role, provided that the six core SDM elements are preserved.

#### Strategies for the implementation of SDM in PPT.

Table 4 presents implementation strategies for PPTs and other interested parties aiming to adopt SDM. These strategies were developed in response to identified barriers and facilitators, using the CFIR-ERIC tool and the Behavior Change Wheel [22,23]. For the inner setting, identifying and preparing champions was included as a strategy to appoint individuals who actively promote, monitor, and support the use of SDM within the team. Leadership engagement and creating a supportive culture were also identified to facilitate ongoing encouragement and feedback. Allocating time for learning and offering structured opportunities to reflect on SDM in team meetings were matched to address limited time and workload barriers.

**Table 4:** Strategies for the implementation of SDM in PPT, based on identified barriers and facilitators.

CFIR domains	Strategy for implementation
I. INNOVATION DOMAIN	
Adaptability of SDM (F)	PPTs: adapt SDM to individual patient needs.
Complexity of SDM (B)	PPTs: participate in SDM training. Use the adapted SDM model for PPT and SDM or goal setting tools.
II. OUTER SETTING DOMAIN	
Policies and laws/finance (B)	*
Partnership and connections (B/F)	PPTs and regional healthcare professionals: establish partnerships and promote network weaving.
III. INNER SETTING DOMAIN	
Physical infrastructure (B/F)	PPTs and parents: establish regular contact moments, for example at school, primary care clinics, or at home.
Work infrastructure (B/F)	Practice managers: provide availability of time for PPTs to become familiar with SDM.
Culture (B/F)	Practice managers and PPTs: develop a practice culture that promotes SDM, and identify and prepare a ‘clinical champion’ within the team (i.e., person who actively supports, promotes, and drives the adoption of SDM).
IV. INDIVIDUALS DOMAIN	
Innovation deliverers (PPTs) – Capability (B/F)	Practice managers: integrate SDM education into staff meetings and facilitate knowledge sharing; PPTs: participate in SDM training and apply environmental restructuring (e.g., prompts, cues, reminders) to stimulate SDM.
Innovation deliverers (PPTs) – Motivation (B/F)	Practice managers: model change, provide supervision, facilitate opportunities for PPTs to shadow colleagues experienced in SDM, identify champions, engage practice leaders, and involve patients in the implementation process.
Innovation recipients (parents and children) – Capability (B/F)	PPTs: educate parents and children in SDM and empower them to take part in decision-making.
Innovation recipients (parents and children) – Motivation (B/F)	Practice managers and PPTs: use environmental restructuring, such as prompts and cues, to stimulate engagement in SDM for parents and children.

CFIR = Consolidated Framework for Implementation Science; F = facilitator; B = barrier; SDM = shared decision-making; PPT = pediatric physical therapist; *No specific strategy was provided for this barrier as finance is part of a broader problem within pediatric physical therapy in the Netherlands, overarching organizations could explore possibilities with health insurances.

Strategies on individual level focused on enhancing the capability, opportunity, and motivation of both PPTs and families. Training and education aim to strengthen PPTs communication and goal-setting skills. The adapted SDM model (Fig 2) can be used as a base for education of PPTs. The strategy ‘model change’ refers to demonstrating desired behavior through leadership and colleagues to encourage adoption of SDM. Providing supervision and opportunities to shadow colleagues allows less experienced PPTs to observe and practice SDM under guidance, promoting learning through modeling and feedback. For families, strategies such as education, empowerment, and environmental cues were selected to enhance understanding of SDM and to increase participation. These include providing accessible information about SDM, prompts or reminder materials, and encouraging active involvement during consultations

## Discussion

SDM is widely recognized as essential for patient-centered care [6,7], but practical guidance for its use in pediatric physical therapy in primary care is lacking. This study explored how and when to apply SDM in this context. Findings show that SDM should begin at intake and goal setting, with ongoing, individualized involvement of both children and parents throughout therapy. When comparing therapy options, treatment frequency, duration, homework, expectations for families and therapists, and home possibilities should be discussed. Based on these findings, we adapted the goal-based SDM model of van der Pol et al. [11] for pediatric physical therapy. Additionally, we identified barriers and facilitators influencing the implementation of SDM. While adaptability of SDM and a supportive practice culture were identified as facilitators, barriers such as time constraints, and the challenge of balancing multiple perspectives persist. Implementation strategies include professional training, use of SDM tools, sufficient contact with parents, taking time to learn SDM, a supportive team culture, and empowering parents and children.

Although several SDM models exist in physical therapy, including those by Hoffmann et al. [8] and Moore and Kaplan [34], these models do not include a separate step for shared goal formulation. In the model of Moore and Kaplan, recommended by Pacheco-Brousseau et al. for adult physical therapy [35], goals are discussed within the second stage alongside treatment options [34], whereas in PPT goals must be established *before* presenting options, as therapy is tailored to goals formulated by children and parents [2,3]. Our qualitative findings also underscored the importance of early shared goal setting. Secondly, the model of van der Pol et al. explicitly includes multiple discussion partners, such as patients and caregivers [11], which is essential within PPT.

Although participants emphasized the relevance of SDM, they mentioned that it has not yet been fully achieved in daily pediatric physical therapy practice. The main identified barriers for its implementation are in line with previous SDM research stating that it is a complex process influenced by beliefs, cognitions, and contextual challenges [36]. Advocating for SDM as the correct moral choice does not necessarily ensure that it will be applied in daily practice [37]. Some PPTs mentioned that training is necessary. For developing proper SDM training, Spinnewijn et al. suggested practice assessments, reflective practice and open discussions on the usefulness of SDM [36]. As goal setting has been recognized as a key aspect of SDM in pediatric physical therapy by our participants, we propose using the adapted goal-based SDM model presented in Fig 2. Although proper training enhances capability of professionals, it does not address other important barriers. Therefore, multifaceted implementation strategies are likely to be most effective [38]. Our strategies presented in Table 4 could be used when implementing SDM in pediatric physical therapy. This aligns with other research indicating strategies such as training of professionals, team-focus on SDM, creating time for SDM by organizational management, feedback on consultations, and patient involvement in the implementation effort [39].

To further facilitate implementation, tools such as decision aids are often used as guidance for SDM [16]. However, these aids are often developed for patients with a specific condition with a discrete set of preference-sensitive options. Within pediatric physical therapy, therapy plans are tailored to personal goals, preferences, and possibilities at home. As a result, developing a decision aid applicable to various situations is challenging. However, generic tools for parents and children, such as ‘three good questions’ could be used to prepare them for SDM [40]. Additionally, as PPTs desired guidance for setting goals, tools such as Goal Attainment Scaling^37^ or the Canadian Occupational Performance Measure^38^ could be used for goal setting. Patient-Reported Outcome Measures (PROMs) can also facilitate goal setting in SDM by providing insight into patient perspectives [41,42]. PROMs, such as the recommended core PROM set for pediatric physical therapy [43], could be used in the preparation phase as well as the goal talk phase.

Time constraints represented a significant barrier according to our participants, also noted in other research [9,17,44]. Although evidence suggests that applying SDM during a consultation does not necessarily require more time [45], less is known about the time needed for therapists to develop and routinely apply SDM skills. A systematic review distinguishes between extra time required for SDM within clinical encounters and the lack of time for SDM due to other tasks [44]. Within the Behavior Change Wheel, limited organizational time reflects a physical opportunity barrier, whereas PPTs perception that SDM is time-consuming appears to relate primarily to a capability barrier associated with learning new skills. For PPTs to effectively implement SDM, they must feel they have sufficient time to develop and apply new skills. We believe that time demands may be higher during early implementation but decrease as PPTs gain experience. Given its benefits, we advise therapists to invest time in mastering SDM and make use of the positive outcomes it provides.

Participants in our study highlighted the importance of individually evaluating and discussing the level of involvement of children in SDM and indicated that children are recommended to be involved as much as possible. However, most research only focuses on the involvement of parents [18]. For example, one SDM model introduced for use in pediatrics, only includes parents and practitioners, without mentioning children [46]. This may be a result of research in children that was mostly conducted in hospital settings where more ‘high-stake’ decisions are made, whereas most healthcare professionals, parents and children were found to be more willing to involve children in ‘low-stake’ decisions [44]. Pediatric physical therapy in primary healthcare does not include life-threatening decisions. Besides that, research shows that children are often capable of identifying their own rehabilitation goals, leading to better engagement and motivation during therapy [47–49].

Although our results indicate that involvement of children is preferable, some participating adolescents and parents mentioned that the start of therapy could lead to a feeling of uncertainty, which led them to place the responsibility of decision-making on the PPT. Similar findings were reported in a study on parent involvement in speech therapy, indicating that parents often feel insecure at the start of therapy, leading to a sense of dependence on the therapist [50]. Empowerment of parents, defined as a combination of ability, motivation, and having time and space for parents to ask questions and share their thoughts, was mentioned as a key focus [50]. Additionally, adolescents and parents in our study preferred to be asked if and how they wanted to be included in making decisions (step three: *choice talk*). This aligns with a systematic review on the roles of parents and professionals in SDM [51]. The authors propose explicit discussions on the roles of parents and professionals to create better alignment with their needs and wishes [51]. Empowerment of children and their parents, and discussions on how parents and children want to be involved may be beneficial in PPT as well, and can be done within step three of the model: *choice talk*. To enable this, the PPT should provide children and parents with sufficient information about the value and process of SDM, to allow a good conversation around mutual expectations, including their role in SDM.

Our study is one of the first to provide guidance on SDM in pediatric physical therapy. We included multiple relevant participant groups in our focus groups and validated the focus group results with a survey among PPTs. However, this study also has some limitations. First, the caregiver group consisted exclusively of mothers, which may have affected the results. Second, this study did not assess participants’ baseline knowledge of SDM or the current state of its implementation. Furthermore, PPTs included in the survey were all participating in a training on SDM and PROMs, which could have introduced bias due to their interest in these topics. Last, extending the survey to parents and adolescents could have been beneficial as well.

To conclude, this study provides valuable insights into applying SDM in pediatric physical therapy through a goal-based SDM model and barriers and facilitators for the implementation including strategies for implementing SDM in pediatric physical therapy in a primary care setting. A multifaceted implementation approach including training of PPTs in SDM, ensuring sufficient contact moments with parents, providing adequate time to become familiar with new approaches, a team culture that promotes SDM, and informing and empowering parents and children could be beneficial. Future research should focus on implementation and evaluation of SDM in primary clinical practice.
